# Goats Follow Human Pointing Gestures in an Object Choice Task

**DOI:** 10.3389/fpsyg.2020.00915

**Published:** 2020-05-19

**Authors:** Christian Nawroth, Zoe M. Martin, Alan G. McElligott

**Affiliations:** ^1^Institute of Behavioural Physiology, Leibniz Institute for Farm Animal Biology, Dummerstorf, Germany; ^2^Biological and Experimental Psychology, School of Biological and Chemical Sciences, Queen Mary University of London, London, United Kingdom; ^3^Department of Life Sciences, Centre for Research in Ecology, Evolution and Behaviour, University of Roehampton, London, United Kingdom

**Keywords:** farm animals, human–animal interaction, livestock, referential information, social cognition

## Abstract

Dogs (*Canis lupus familiaris*) are extremely adept in interpreting human-given cues, such as the pointing gesture. However, the underlying mechanisms on how domestic non-companion species use these cues are not well understood. We investigated the use of human-given pointing gestures by goats (*Capra hircus*) in an object choice task, where an experimenter surreptitiously hid food in one of two buckets. Subjects first had to pass a pre-test where the experimenter indicated the location of the food to the subject by a proximal pointing gesture. Subjects that succeeded in the use of this gesture were transferred to the actual test. In these subsequent test trials, the experimenter indicated the location of the food to the subject by using three different pointing gestures: proximal pointing from a middle position (distance between target and index finger: 30 cm), crossed pointing from the middle position (distance between target and index finger: 40 cm), asymmetric pointing from the position of the non-baited bucket (distance between target and index finger: 90 cm). Goats succeeded in the pointing gestures that presented an element of proximity (proximal and crossed) compared to when the experimenter was further away from the rewarded location (asymmetric). This indicates that goats can generalize their use of the human pointing gesture but might rely on stimulus/local enhancement rather than referential information. In addition, goats did not improve their responses over time, indicating that no learning took place. The results provide a greater understanding of human–animal interactions and social-cognitive abilities of farm animals, which allows for the provision of enhanced management practices and welfare conditions.

## Introduction

Via their domestication as a companion animal, dogs are extra-ordinarily adapted to living in an anthropogenic environment and to communicate with humans. Dogs, like children, establish attachment bonds with humans (Rehn et al., 2013), refer to humans when confronted with an unsolvable problem (Miklósi et al., 2003), socially learn from humans in a spatial learning task (Pongrácz et al., 2001), and can use human pointing gestures to gather information about their environment (Kaminski and Nitzschner, 2013).

Increased performance in using a human pointing gesture is one of the most prominent outcomes of domestication and is often tested in a so-called object choice task. Here, an experimenter hides food out of sight of a test subject under one of two or three cups. After baiting the cup, the experimenter indicates the location of the food to the subject by a pointing gesture. In this task, dogs cannot only locate the correct location when the gesture is administered in proximity to the correct location. They can also find food rewards located at a significant distance away from the gesturing experimenter (Hare et al., 1998; Lakatos et al., 2009). Additionally, dogs are also able to use novel, unfamiliar cues in which they have had no previous training or exposure (Soproni et al., 2002; Riedel et al., 2008). These results indicate that dogs understand human pointing as a referential signal and do not solely rely on learning and potential enhancement effects (Kaminski and Nitzschner, 2013).

But dogs are not the only species that are able to use human pointing gestures to locate a reward. A number of non-domestic species have also been found to use a human pointing gesture: gray seals, *Halichoerus grypus* (Shapiro et al., 2003), African fur seals, *Arctocephalus pusillus* (Scheumann and Call, 2004), bottlenose dolphins, *Tursiops truncatus* (Xitco et al., 2001) and jackdaws, *Corvus monedula* (von Bayern and Emery, 2009). Regarding other domestic animals, pigs (*Sus scrofa*), and horses (*Equus caballus*) have also been tested in this paradigm (McKinley and Sambrook, 2000; Proops et al., 2010; Nawroth et al., 2014). While most results for these domestic species are positive (but see Gerencsér et al., 2019), it is complicated to assess the actual mechanisms at work when interpreting animals’ use of these gestures. Almost all studies in domestic non-canid species used a standard pointing gesture, administered in a sustained, but sometimes also momentary, manner with the experimenter stretching out it’s ipsilateral hand and being positioned in the middle between both locations. This makes it prone to alternative, more simplistic explanations regarding the mechanism at work: the hand of the experimenter is always closer to the correct rather than the incorrect location (effect of stimulus/local enhancement) and ipsilateral pointing might be a gesture that is frequently employed by humans in daily interactions with animals (effects of learning). More complex gestures, such as cross-pointing with the contralateral arm, or configural positions of the experimenter, such as placing the experimenter behind the incorrect location, have rarely been investigated in domestic non-canid animals (but see for pigs: Nawroth et al., 2014).

Goats, a species primarily domesticated for products such as meat and milk rather than companionship, have been shown to interact with humans in similar ways to dogs in common test paradigms. When confronted with an unsolvable problem, they show frequent audience-dependent gazing and gaze alternations toward a human experimenter (Nawroth et al., 2016b; Langbein et al., 2018). Goats also improved their performance in a spatial learning task by observing a human demonstrator prior to the test itself (Nawroth et al., 2016a). They are also able to use human pointing gestures, but not the head orientation of an experimenter, to locate a reward in an object choice task (Kaminski et al., 2005; Nawroth et al., 2015). However, the gestures displayed in both experiments were administered with the ipsilateral hand and while the experimenter was positioned in the middle of both locations. It is thus not clear whether goats (or other domestic non-companion species) can generalize this skill to other pointing gestures and/or whether their performance in this task is simple due to stimulus/local enhancement effects. Given the lack of research on domestic non-companion species regarding their use of human pointing gestures, the presumed underlying mechanisms in these species to use these cues are thus not well understood.

To extend our knowledge on the use of human pointing gestures in domestic non-companion animals, we investigated the use of human-given pointing gestures by goats in an object choice task. We extended the administered repertoire of pointing gestures used in previous experiments on goats to infer whether they generalize between cues and whether they, to some degree, understand their referential nature. Goats were first tested on a proximal pointing gesture (pre-test). Afterwards, they were additionally confronted with a condition that differed in appearance and was displayed at a similar distance to the target (testing for generalization of pointing gesture), and a condition that looked similar to the initial proximal pointing gestures but was administered from an increased distance to the target (testing for comprehension of referentiality). If goats are solely relying on stimulus/local enhancement, we would predict that they would be able to solve the conditions with the proximal distance to the rewarded location, while they would fail to solve the task with an increased distance. Alternatively, if goats would be able to use the referential information from the pointing gesture, we would expect them to solve all three conditions.

## Animals, Materials, and Methods

### Ethics Statement

Animal care and all experimental procedures were in accordance with the ASAB/ABS Guidelines for the Use of Animals in Research (Association for the Study of Animal Behaviour, 2016). The study was approved by the Animal Welfare and Ethical Review Board committee of Queen Mary University of London (Ref. QMULAWERB072016). All measurements were non-invasive, and the experiment lasted no more than 15 min for each individual goat. If the goats had become stressed, the test would have been stopped.

### Subjects and Housing

The study was carried out at Buttercups Sanctuary for Goats, United Kingdom^1^. A total of 20 goats, which included 13 neutered male and seven female goats of various breeds and ages, were used (Table 1). Goats were fully habituated to human presence and the test arena because of previous research (Baciadonna et al., 2016; Nawroth et al., 2016b). Routine care of the animals was provided by sanctuary employees and volunteers. The goats had *ad libitum* access to hay and were not food restricted before testing. Subjects were tested from 11:00 to 16:00 during August 2016.

**TABLE 1:** Names, sex, age and breed of the twenty goats that participated.

Name	Sex	Age	Breed	Participation in test
Annie	Female	3	Boer	Yes
Dingle	Male	5	Mix	Yes
Gilbert	Male	11	Pygmy	Yes
Jimmy	Male	8	Pygmy	Yes
Leo	Male	4	Pygmy	Yes
Pooky	Female	4	Pygmy	Yes
Ralph	Male	4	Pygmy	Yes
Vern	Male	6	British Toggenburg Mix	Yes
Sticky	Male	7	Mix	Yes
Archie	Male	10	Pygmy	No, did not reach criterion
Cicero	Male	5	Anglo Nubian	No, did not reach criterion
Hattie	Female	4	British Toggenburg X Pygmy	No, did not reach criterion
Marnie	Female	3	Pygmy	No, did not reach criterion
Rodney	Male	9	Pygmy	No, did not reach criterion
Roland	Male	8	Mix	No, did not reach criterion
Sandy	Female	17	Pygmy	No, did not reach criterion
Heidi	Female	5	British Toggenburg	No, lacked motivation
Nadia	Female	6	British Saanen	No, lacked motivation
Rupert	Male	6	British Toggenburg	No, lacked motivation
Wilfred	Male	5	Anglo Nubian	No, lacked motivation

### Experimental Procedure

The research consisted of two stages: a pre-test session and two test sessions, each administered on separate days. For both, each goat was separated for no longer than 15 min in a large, fenced arena (length: 700 cm, width: 530 cm). The tested subject was always able to maintain olfactory and auditory contact with conspecifics. The main experimenter and an assistant who handled the goats were also present within the arena with the test goat. The experimenter was seated on a small, plastic table at one end of the arena and the assistant was positioned at the opposite end of the arena approximately 350 cm away holding the test subject on a leash at the start point. Two red buckets (height: 25 cm, diameter: 25 cm) were positioned on either side of the experimenter, approximately 200 cm apart, in which a food reward (a piece of uncooked pasta) was placed into one of the buckets before a trial started. The pointing gesture was always directed at the bucket that was baited with the food reward.

#### Pre-test

In the pre-test, the goats (*N* = 20) were exposed to a proximal pointing gesture (Table 2). The location of the food reward was alternated between both sides and was for no more than two consecutive trials on the same side. Before the pre-test began, the test goat was exposed to two training trials. The goat was shown the reward being placed into one of the buckets and was then allowed to retrieve the food, which familiarized the subjects with the buckets. Before each pre-test trial started, the experimenter placed both hands into each bucket simultaneously during baiting so as not to indicate the location of the food reward to the goat. A trial started when the assistant released the goat from the start point. The experimenter pointed at the bucket that contained the food reward at a maximum of five times in a dynamic manner. When the goat approached within approximately 1.5 m of either bucket, the experimenter stopped the dynamic gesture and displayed a sustained pointing gesture toward the rewarded bucket. To accompany the pointing gesture, the experimenter also alternated her head orientation between the subject and the bucket to further reinforce the communicative nature of the cue. Each goat received two training trials and a maximum of 16 trials in the pre-test, all administered on one day. If a goat chose the baited bucket in six consecutive pre-test trials (binomial test, *P* = 0.031), it proceeded to the test.

**TABLE 2:** The three pointing gestures plus the control condition that were administered to the goats in the pre-test and test trials.

Condition	Description
Proximal (pre-test and test)	The experimenter dynamically pointed at the bucket containing the food reward until the goat approached either of the two buckets. When the goat approached within approximately 1.5 m of either bucket, the experimenter stopped the dynamic gesture and displayed a sustained pointing gesture toward the rewarded bucket. The baited bucket was positioned approximately 30 cm away from the tip of the experimenter’s finger when the arm was fully stretched
Crossed (test)	The same as the proximal gesture (including preceding dynamic pointing) but the experimenter pointed across her body to the bucket with the food reward on the opposite side of her body. The baited bucket was positioned approximately 40 cm away from the tip of the experimenter’s finger when the arm was fully stretched
Asymmetric (test)	The same as the proximal gesture (including preceding dynamic pointing) but experimenter sat behind the bucket that did not contain the food reward and pointed across to the bucket that was baited with the food reward. The baited bucket was positioned approximately 90 cm away from the tip of the experimenter’s finger when the arm was fully stretched
Control (test)	The experimenter sat motionless with her hands behind her back and was facing the goat

#### Test

**FIGURE 1:**
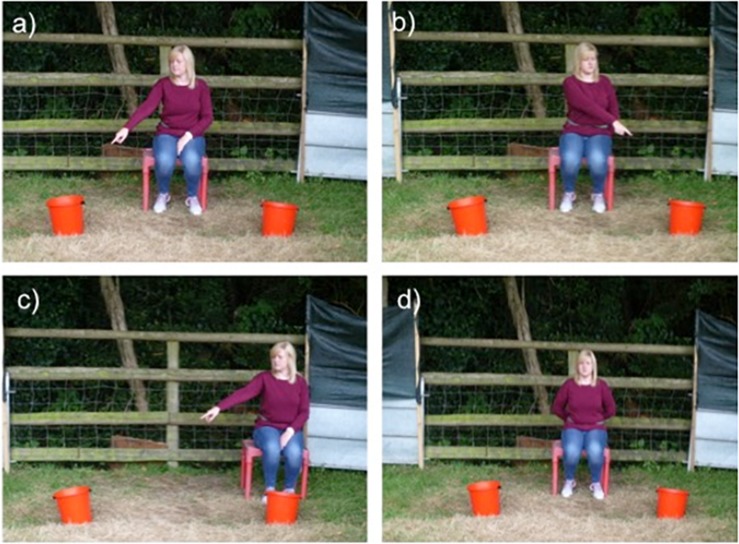
Images of the four test conditions: **(a)** proximal (the whole arm is visibly pointing at the rewarded bucket), **(b)** crossed (the arm is pointing at the rewarded bucket, but only the wrist and hand are clearly visible), **(c)** asymmetric (the whole arm is visibly pointing at the bucket, while the experimenter is positioned behind the non-rewarded bucket), and **(d)** control.

Procedure for test trials was similar to that of the pre-test trials with the exception that goats (*N* = 9; two females, seven males) were exposed to four different conditions: proximal pointing, crossed pointing, asymmetric pointing and a control condition (Table 2 and Figures 1a–d). In all conditions, excluding the control condition, the experimenter pointed at the bucket that contained the food reward at a maximum of five times in a dynamic manner. When the goat approached within approximately 1.5 m of either bucket, the experimenter stopped the dynamic gesture and displayed a sustained pointing gesture toward the rewarded bucket. To accompany the three pointing gestures, the experimenter also alternated her head orientation between the subject and the bucket. Test trials started three days after the pre-test and were administered over two sessions (one per day) including 16 trials each. Identical to the pre-test, each test session started with two motivation trials where the goat was shown the food reward being placed in either bucket (left–right or right–left). In the test trials, each of the four conditions was presented to the goat four times pseudo-randomly within the 16 trials of each session and was not presented more than twice in a row. The location of the food reward was also alternated and pseudo-randomly balanced between both sides and was for no more than two consecutive trials on the same side.

#### Data Coding and Analysis

A digital video camera (Sony HCR-CX 190E Camcorder) was used to record the trials, which was placed on a tripod and positioned behind the fence where the experimenter was seated. We scored which bucket (correct or incorrect) the test subject chose for each trial. Choice was defined as physical contact of the goat with the bucket. If a goat needed more than 60 s to indicate a choice, the trial was scored as “no choice.” We also scored if goats approached the index finger of the experimenter before making a choice (from physical contact to 5 cm distance between finger and goat). The latter was done to assess whether goats were only attracted to the hand movement of the experimenter, rather than the pointing direction itself. To assess inter-observer reliability, 50% of the videos were coded by a second coder unfamiliar to the initial hypothesis. Inter-observer reliability for choice analysis (Cohen’s κ = 0.972, *P* < 0.0001) showed a very high level of agreement. Statistical analyses were carried out in R v.3.6 (R Core Team, 2017). The choice behavior of goats in the test trials was treated as a binary variable (choose correct bucket = 1, choose incorrect bucket = 0) and was analyzed with a generalized mixed-effects model fit with binomial family distribution and logit link function (GLMM; glmer function, lme4 library; Pinheiro and Bates, 2000). “Condition” (factor with four levels: proximal, crossed, asymmetric, control) and “Session” (factor with two levels: 1, 2) as well as their interaction were included as fixed factors. The statistical significance of the factors was assessed by comparing the models with and without the factor included. *P*-values were calculated using likelihood ratio tests (LRT) and when a significant effect of “Condition” was detected, we carried out Tukey *post hoc* tests (glht function, multcomp library, Hothorn et al., 2008). Identity of the goats was included as a random factor to control for repeated measurements. To analyze whether the group performance in each condition deviated from random chance level (i.e., 4 out of 8 trials correct) we used one-sample *t*-tests. Goats rarely approached the index finger of the experimenter when one of the three pointing gestures were administered (in 12 out of 216 test trials, excluding the control condition) so we only provide descriptive statistics on this factor. All tests were two-tailed, and the alpha level was set at 0.05 for all statistical tests. An example video, as well as raw data and code can be found in the Supplementary Material and here: https://osf.io/vy5md/.

## Results

### Pre-test

Of the 20 goats that participated in the pre-test, nine goats advanced to the test trials (mean ± SD: 9.33 ± 3.2 sessions). Seven goats completed the 16 pre-test trials but did not reach the criterium and were thus excluded. Four additional goats stopped participating due to a lack of motivation.

### Test

**FIGURE 2:**
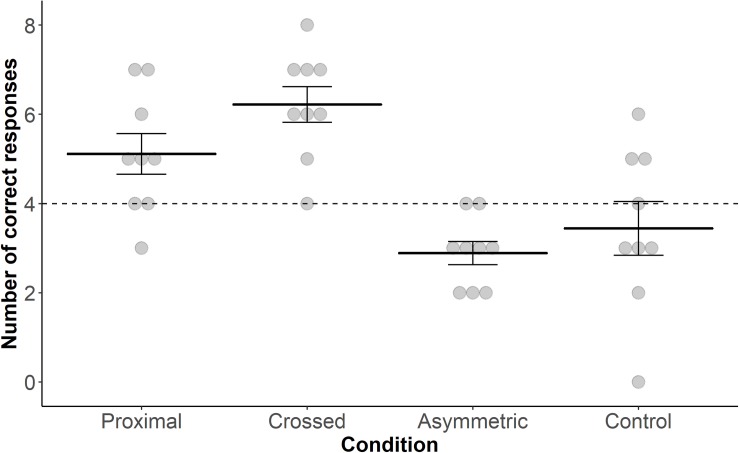
Dot plot including mean performance and standard errors over the four test conditions: proximal, crossed, asymmetric and control. Filled dots represent individual data points. The dashed line represents chance level (i.e., 4 out of 8 trials correct).

Goat performance in locating the correct bucket in the task differed significantly across conditions (GLMM: *n* = 288 trials, 9 goats; X ^2^ = 33.143, *P* = 0.001; Figure 2). Neither “Session” nor an interaction between “Condition” and “Session” was found (“Session”: X ^2^ = 0.774, *P* = 0.37; interaction: X ^2^ = 0.489, *P* = 0.92). *Post hoc* Tukey tests revealed that the goats chose the correct bucket more often in response to the proximal pointing gesture compared to the asymmetric pointing gesture (*z* = 3.293, *P* = 0.006) and tended to do so compared to the control condition (*z* = 2.490, *P* = 0.06). They also chose the correct bucket more often in response to the crossed pointing gesture in comparison to the asymmetric pointing gesture (*z* = 4.869, *P* < 0.001) and the control condition (*z* = 4.145, *P* < 0.001). All other comparisons were not significantly different.

Performance in the conditions “proximal,” “crossed,” and “asymmetric” differed significantly from chance level (i.e., 50% success rate; proximal, *t*_8_ = 2.443, *P* = 0.04; crossed, *t*_8_ = 5.547, *P* < 0.001; asymmetric, *t*_8_ = −4.264, *P* = 0.003; one-sample *t*-test); this was not the case for the control condition (*t*_8_ = −0.921, *P* = 0.384).

Most goats (6/9) approached the experimenter’s hand/finger in one or more test trials before choosing either bucket. However, in total, they only approached the hand/finger in 12 out of 216 test trials (5.55%, excluding the trials of the control condition). In 10 out of these 12 trials, the test subject chose the baited bucket.

## Discussion

We investigated the use of different human pointing gestures by goats in an object choice task. Goats succeeded in following the pointing gestures that presented an element of proximity (proximal and crossed) compared to when the experimenter was further away from the rewarded location (asymmetric). This indicates that goats can generalize over pointing gestures but might not be able to use the referential information conveyed in those gestures (Miklósi and Soproni, 2006; Krause et al., 2018).

Goats performed well when confronted with the proximal and the crossed pointing gestures, but not in the asymmetric condition. The first two gestures included a decreased distance between the index finger of the experimenter and the rewarded bucket, compared to the asymmetric condition. This indicates that stimulus/local enhancement and/or positive reinforcement to approach a human hand (or the human itself) might best explain the good performance in the proximal and crossed condition (Krueger et al., 2011; Bensoussan et al., 2016). However, the low direct approaches to the experimenter’s finger indicate that goats did not show increased interest to physically interact with the experimenter *per se*.

Goats in our study approached the bucket that was indicated by a human pointing gesture in the asymmetric condition significantly less likely compared to chance level, indicating that they were attracted by the experimenter positioned at the location of the incorrect location. However, we cannot completely rule out that goats might use referential information in this context, as the stimulus/local enhancement by the human positioned behind the incorrect location might have overridden any effect of it. Other test designs, such as an experimenter, placed in the middle, having two cups at an increased distance in front of them (Lakatos et al., 2009) should thus be implemented.

We did not find that goats’ performance improved over the two sessions, indicating a lack of learning. They were also not able to locate the hidden reward in the control condition, indicating no inadvertent cueing during the test procedure. Four of the initial 20 goats (20%) lost motivation to participate in the pre-test and were subsequently excluded. This might be due to distractions in the environment or fatigue. While a dropout rate of approximately 20% can be considered the norm in object-choice tasks (Kaminski and Nitzschner, 2013), another seven subjects did not reach the criterion to proceed to the pre-test. The exclusion of these subjects in the test might have skewed group performance toward higher numbers. However, not reaching the criterion does not equal that goats were not able to follow the pointing gesture. In fact, six out of the seven subjects that did not reach the criterion choose the rewarded bucket in nine or more of the 16 administered trials in the pre-test. In the future, it would be interesting to test other populations of goats with different backgrounds regarding their interactions with humans. Goats in our study lived at a sanctuary and experience daily positive interactions (e.g., feeding and grooming) with familiar and unfamiliar humans. Testing goats of different ages, as well as feral or wild goats, will shed light on the origin of domestic goats’ ability to use pointing gestures by humans.

## Data Availability Statement

All datasets generated for this study are included in the article/Supplementary Material.

## Ethics Statement

The animal study was reviewed and approved by the Animal Welfare and Ethical Review Board committee of Queen Mary University of London. Written informed consent was obtained from the owners for the participation of their animals in this study. Written informed consent was obtained from the individuals and/or minors’ legal guardian/next of kin for the publication of any potentially identifiable images or data included in this article.

## Author Contributions

CN and AM designed the experiment. CN and ZM collected and analyzed the data. All authors wrote the manuscript and approved it for publication.

## Conflict of Interest

The authors declare that the research was conducted in the absence of any commercial or financial relationships that could be construed as a potential conflict of interest.

## Footnotes

1 http://www.buttercups.org.uk

## References

[B1] Association for the Study of Animal Behaviour (2016). Guidelines for the treatment of animals in behavioural research and teaching. *Anim. Behav.* 111 1–9. 10.1006/anbe.2000.1652 11170716

[B2] Baciadonna L. Nawroth C. McElligott A. G. (2016). Judgement bias in goats (*Capra hircus*): investigating the effects of human grooming. *PeerJ* 4:e2485. 10.7717/peerj.2485 27761311 PMC5068416

[B3] Bensoussan S. Cornil M. Meunier-Salaün M.-C. Tallet C. (2016). Piglets learn to Use combined human-given visual and auditory signals to find a hidden reward in an object choice task. *PLoS One* 11:e0164988. 10.1371/journal.pone.0164988 27792731 PMC5085045

[B4] Gerencsér L. Pérez Fraga P. Lovas M. Újváry D. Andics A. (2019). Comparing interspecific socio-communicative skills of socialized juvenile dogs and miniature pigs. *Anim. Cogn.* 22 917–929. 10.1007/s10071-019-01284-z 31256339 PMC6834752

[B5] Hare B. Call J. Tomasello M. (1998). Communication of food location between human and dog (*Canis Familiaris*). *Evol. Commun.* 2 137–159. 10.1075/eoc.2.1.06har

[B6] Hothorn T. Bretz F. Westfall P. (2008). Simultaneous inference in general parametric models. *Biometrical J.* 50 346–363. 10.1002/bimj.200810425 18481363

[B7] Kaminski J. Nitzschner M. (2013). Do dogs get the point? A review of dog–human communication ability. *Learn. Motiv.* 44 294–302. 10.1016/j.lmot.2013.05.001

[B8] Kaminski J. Riedel J. Call J. Tomasello M. (2005). Domestic goats, *Capra hircus*, follow gaze direction and use social cues in an object choice task. *Anim. Behav.* 69 11–18. 10.1016/j.anbehav.2004.05.008

[B9] Krause M. A. Udell M. A. R. Leavens D. A. Skopos L. (2018). Animal pointing: Changing trends and findings from 30 years of research. *J. Comp. Psychol.* 132 326–345. 10.1037/com0000125 29952588

[B10] Krueger K. Flauger B. Farmer K. Maros K. (2011). Horses (*Equus caballus*) use human local enhancement cues and adjust to human attention. *Anim. Cogn.* 14 187–201. 10.1007/s10071-010-0352-7 20845052

[B11] Lakatos G. Soproni K. Dóka A. Miklósi Á (2009). A comparative approach to dogs’ (*Canis familiaris*) and human infants’ comprehension of various forms of pointing gestures. *Anim. Cogn.* 12 621–631. 10.1007/s10071-009-0221-4 19343382

[B12] Langbein J. Krause A. Nawroth C. (2018). Human-directed behaviour in goats is not affected by short-term positive handling. *Anim. Cogn.* 21 795–803. 10.1007/s10071-018-1211-1 30173331

[B13] McKinley J. Sambrook T. D. (2000). Use of human-given cues by domestic dogs (*Canis familiaris*) and horses (*Equus caballus*). *Anim. Cogn.* 3 13–22. 10.1007/s10071-009-0257-5 19588176

[B14] Miklósi Á Kubinyi E. Topál J. Gácsi M. Virányi Z. Csányi V. (2003). A simple reason for a big difference: wolves do not look back at humans, but dogs do. *Curr. Biol.* 13 763–766. 10.1016/s0960-9822(03)00263-x 12725735

[B15] Miklósi Á Soproni K. (2006). A comparative analysis of animals’ understanding of the human pointing gesture. *Anim. Cogn.* 9 81–93. 10.1007/s10071-005-0008-1 16235075

[B16] Nawroth C. Baciadonna L. McElligott A. G. (2016a). Goats learn socially from humans in a spatial problem-solving task. *Anim. Behav.* 121 123–129. 10.1016/j.anbehav.2016.09.004

[B17] Nawroth C. Brett J. M. McElligott A. G. (2016b). Goats display audience-dependent human-directed gazing behaviour in a problem-solving task. *Biol. Lett.* 12:20160283. 10.1098/rsbl.2016.0283 27381884 PMC4971169

[B18] Nawroth C. Ebersbach M. von Borell E. (2014). Juvenile domestic pigs (*Sus scrofa domestica*) use human-given cues in an object choice task. *Anim. Cogn.* 17 701–713. 10.1007/s10071-013-0702-3 24197275

[B19] Nawroth C. von Borell E. Langbein J. (2015). ‘Goats that stare at men’: dwarf goats alter their behaviour in response to human head orientation, but do not spontaneously use head direction as a cue in a food-related context. *Anim. Cogn.* 18 65–73. 10.1007/s10071-014-0777-5 24997158

[B20] Pinheiro J. C. Bates D. M. (2000). *Mixed-Effects Models in S and S-PLUS.* New York, NY: Springer-Verlag.

[B21] Pongrácz P. Miklosi A. Kubinyi E. Gurobi K. Topal J. Csányi V. (2001). Social learning in dogs: the effect of a human demonstrator on the performance of dogs in a detour task. *Anim. Behav.* 62 1109–1117. 10.1006/anbe.2001.1866

[B22] Proops L. Walton M. McComb K. (2010). The use of human-given cues by domestic horses, *Equus caballus*, during an object choice task. *Anim. Behav.* 79 1205–1209. 10.1007/s10071-009-0257-5 19588176

[B23] R Core Team (2017). *R: A Language and Environment for Statistical Computing.* Vienna: R Core Team.

[B24] Rehn T. McGowan R. T. S. Keeling L. J. (2013). Evaluating the strange situation procedure (SSP) to assess the bond between dogs and humans. *PLoS One* 8:56938. 10.1371/journal.pone.0056938 23437277 PMC3577677

[B25] Riedel J. Schumann K. Kaminski J. Call J. Tomasello M. (2008). The early ontogeny of human - dog communication. *Anim. Behav.* 75 1003–1014. 10.1016/j.anbehav.2007.08.010

[B26] Scheumann M. Call J. (2004). The use of experimenter-given cues by South African fur seals (*Arctocephalus pusillus*). *Anim. Cogn.* 7 224–230. 10.1007/s10071-004-0216-0 15057598

[B27] Shapiro A. D. Janik V. M. Slater P. J. B. (2003). A gray seal’s (*Halichoerus grypus*) responses to experimenter-given pointing and directional cues. *J. Comp. Psychol.* 117 355–362. 10.1037/0735-7036.117.4.355 14717636

[B28] Soproni K. Miklósi Á Topál J. Csányi V. (2002). Dogs’ (*Canis familaris*) responsiveness to human pointing gestures. *J. Comp. Psychol.* 116 27–34. 10.1037/0735-7036.116.1.27 11926681

[B29] von Bayern A. M. P. Emery N. J. (2009). Jackdaws respond to human attentional states and communicative cues in different contexts. *Curr. Biol.* 19 602–606. 10.1016/j.cub.2009.02.062 19345101

[B30] Xitco M. J. Gory J. D. Kuczaj S. A. (2001). Spontaneous pointing by bottlenose dolphins (*Tursiops truncatus*). *Anim. Cogn.* 4 115–123. 10.1007/s100710100107

